# Supplementary material for: Subcontinental Genetic Diversity in the All of Us Research Program: Implications for Biomedical Research
Source: bioRxiv. 2025 Jan 10:2025.01.09.632250. Preprint. [Version 1] doi: 10.1101/2025.01.09.632250 (PMC11741438; doi:10.1101/2025.01.09.632250)
Supplement: Supplement 1 [file media-1.pdf]

Supplementary Figures

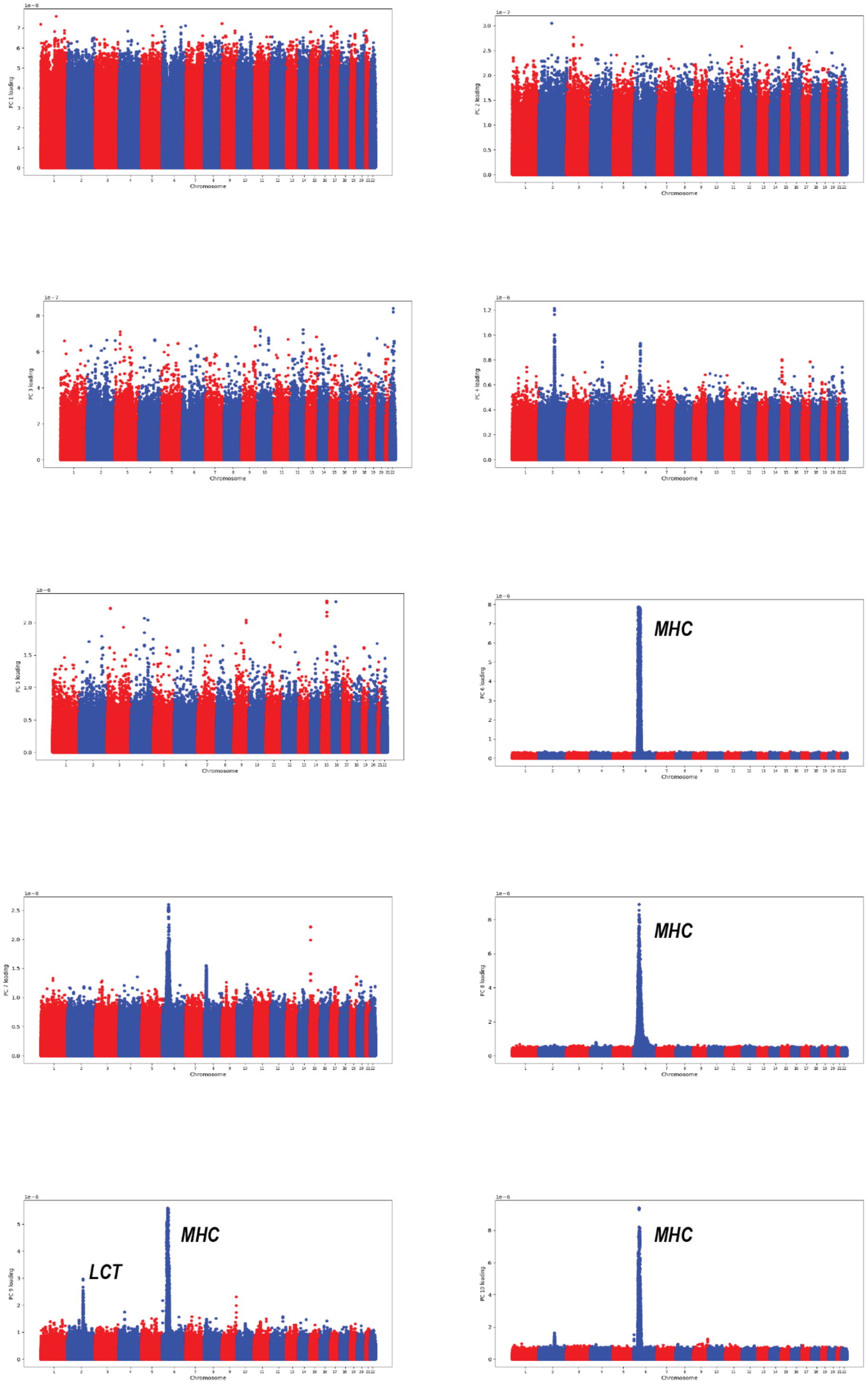

Fig. S1. Manhattan plot of SNP loadings for the top 10 PCs from the unsupervised PCA analysis of *All of* Us. *MHC,* Major Histocompatibility Complex. *LCT*, lactase.

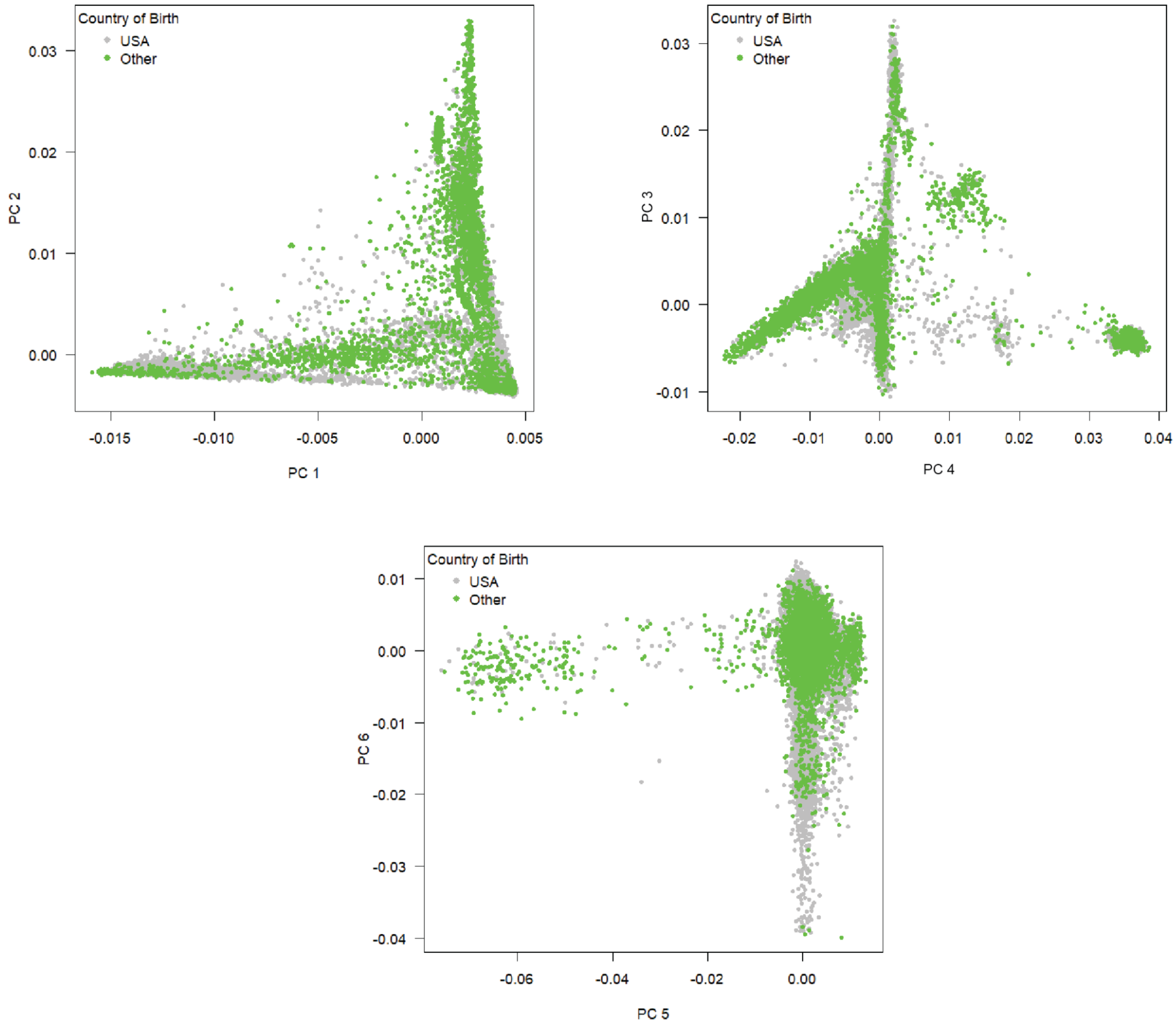

Fig. S2. Genetic diversity of *All of Us* participants stratified by country of birth. The *All of Us* workbench provides binary information on participants' country of birth, categorized as either "United States" or "non-United States".

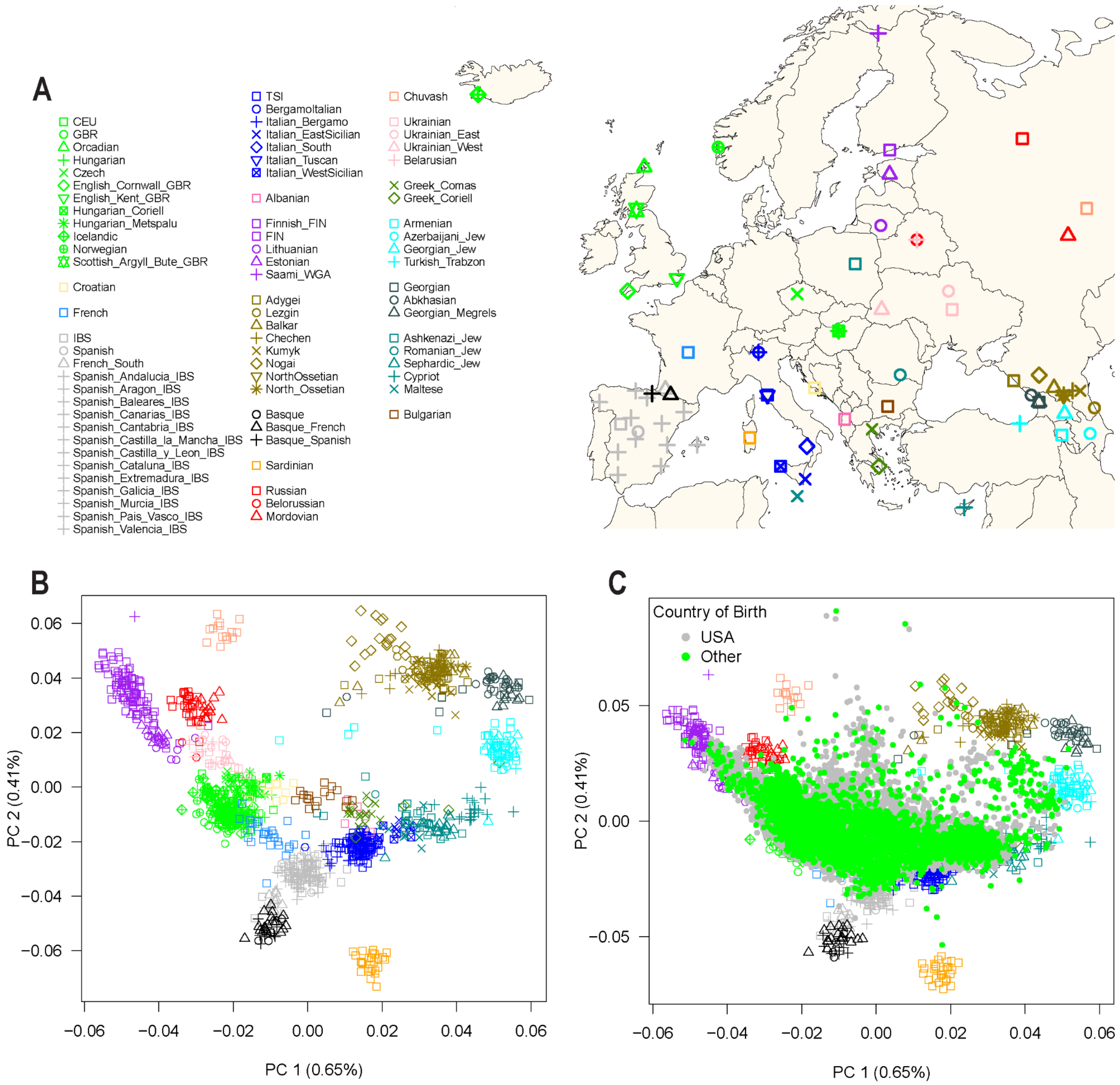

Fig. S3. European reference panels and coverage of European genetic diversity by *All of Us* participants self-identified as "White". A) Map of Europe showing the geographic location of samples from 79 European populations. The map was drawn using the R package "maps" version 3.4.1. B) The first two principal components (PCs 1 and 2) of genetic diversity and the percent variance explained. C) Projection of *All of Us* participants self-identifying as "White" onto the European reference panel. This figure is adapted from Gouveia *et al.* 2023 (PMID: 37935687).

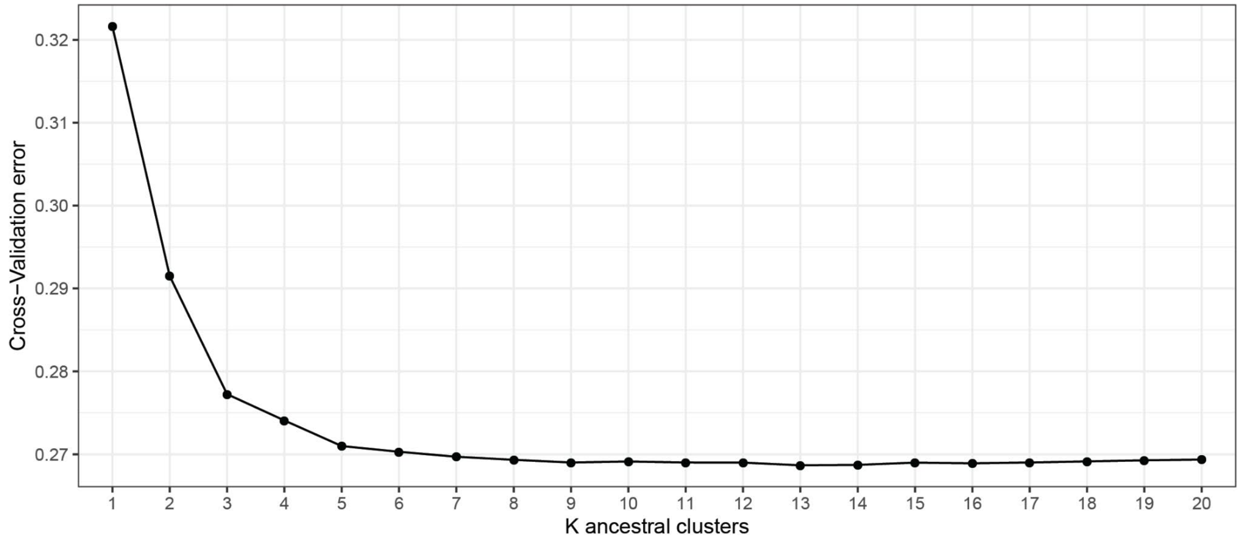

Fig. S4. Cross-validation plot supporting K = 13 as the most likely number of ancestral components.
